# Structural Analyses of the Slm1-PH Domain Demonstrate Ligand Binding in the Non-Canonical Site

**DOI:** 10.1371/journal.pone.0036526

**Published:** 2012-05-04

**Authors:** Kanchan Anand, Kenji Maeda, Anne-Claude Gavin

**Affiliations:** 1 European Molecular Biology Laboratory Heidelberg, Structural and Computational Biology Unit, Heidelberg, Germany; 2 Max-Planck-Institut für Molekulare Physiologie, Abteilung Physikalische Biochemie, Dortmund, Germany; Institute of Enzymology of the Hungarian Academy of Science, Hungary

## Abstract

**Background:**

Pleckstrin homology (PH) domains are common membrane-targeting modules and their best characterized ligands are a set of important signaling lipids that include phosphatidylinositol phosphates (PtdInsPs). PH domains recognize PtdInsPs through two distinct mechanisms that use different binding pockets on opposite sides of the β-strands 1 and 2: i) a canonical binding site delimited by the β1-β2 and β3-β4loops and ii) a non-canonical binding site bordered by the β1-β2 and β5-β6loops. The PH domain-containing protein Slm1 from budding yeast *Saccharomyces cerevisiae* is required for actin cytoskeleton polarization and cell growth. We recently reported that this PH domain binds PtdInsPs and phosphorylated sphingolipids in a cooperative manner.

**Principal Findings:**

To study the structural basis for the Slm1-PH domain (Slm1-PH) specificity, we co-crystallized this domain with different soluble compounds that have structures analogous to anionic lipid head groups of reported Slm1 ligands: inositol 4-phosphate, which mimics phosphatidylinositol-4-phosphate (PtdIns(4)P), and phosphoserine as a surrogate for dihydrosphingosine 1-phosphate (DHS1-P). We found electron densities for the ligands within the so-called non-canonical binding site. An additional positively charged surface that contacts a phosphate group was identified next to the canonical binding site.

**Conclusions:**

Our results suggest that Slm1-PH utilizes a non-canonical binding site to bind PtdInsPs, similar to that described for the PH domains of β-spectrin, Tiam1 and ArhGAP9. Additionally, Slm1-PH may have retained an active canonical site. We propose that the presence of both a canonical and a non-canonical binding pocket in Slm1-PH may account for the cooperative binding to PtdInsPs and DHS-1P.

## Introduction

**Figure 1:**
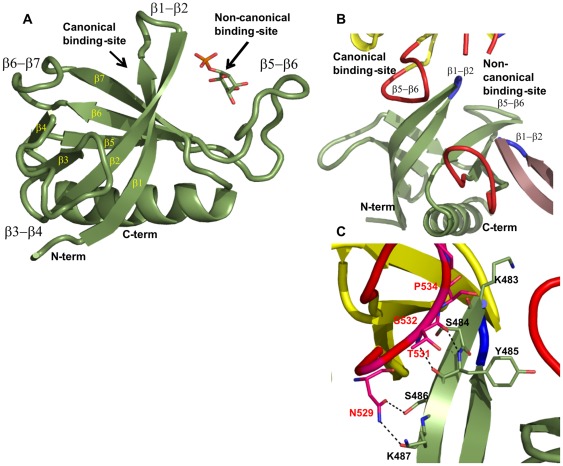
Overall structure of the Slm1-PH domain. (**A**) Ribbon representation of the 1.76 Å structure (green) showing that Slm1-PH folds into a seven-stranded β-sheet terminated with an α-helix. All secondary structure elements are labeled in yellow and black. Both canonical and non-canonical binding sites are also marked. (**B**) In the X-ray crystal structure the canonical binding site is partially occluded by the β5-β6 loop (in red) of the neighboring molecules (in yellow and violet) on both sides. The β1-β2 loops of all the molecules are shown in blue. All the loops in Figure 1B have been smoothened using PyMOL for clarity. (**C**) Detailed view of the interactions between the β1-β2 loop and the β5-β6 loop of neighboring molecules. Primarily, the side-chain residues of the β5-β6 loop are making contact with the main-chain atoms of the β1-β2 loop region.

Pleckstrin homology (PH) domains are short segments of approximately 120 amino acids and are present in a wide range of proteins. Despite their poor conservation at the sequence level, PH domains share a common fold that consists of a seven-stranded antiparallel β-sheet and a C-terminal amphipathic α-helix (Figure 1A). The human genome and that of the yeast *Saccharomyces cerevisiae* encode at least 252 and 27 proteins, respectively, that contain one or several PH domains and these are involved in a wide range of biological processes, including cellular signaling, cytoskeleton organization, membrane trafficking and lipid metabolism [1], [2].

The best-characterized ligands for PH domains are phosphatidylinositol phosphates (PtdInsPs) [1], [3], [4], [5], [6], [7], [8], [9], [10], [11] and binding to this important class of signaling lipid contributes to protein recruitment to cellular membranes. The ligand specificity of each PH domain is variable. For example, the PH domain of phospholipase Cδ (PLCδ) binds phosphatidylinositol-4,5-bisphosphates (PtdIns(4,5)P_2_) with high affinity (K_D_ = 2 µM [1]), whereas the affinity of the dynamin 1 PH domain for the same phospholipid is in the millimolar range. The protein kinase B (b/akt) PH domain, on the other hand, binds PtdIns(3,4,5)P_3_ and PtdIns(3,4)P_2_ with similar affinities, but does not bind to PtdIns(3)P or PtdIns(3,5)P_2_. However, only a minority of PH domains actually target PtdInsPs with high specificities and affinities and in fact, the vast majority of PH domains either poorly discriminate between different PtdInsPs or do not bind lipids at all [12], [13], [14], [15]. Accordingly, some PH domains have been proposed to mediate protein-protein interactions or to bind proteins and lipids through cooperative mechanisms [16].

Both X-ray crystallography and nuclear magnetic resonance spectroscopy (NMR) have been used to understand the structural determinants of lipid recognition by PH domains. In general, head groups of PtdInsPs are accommodated on a positively charged surface of PH domains and two main binding modes have been described to date (Figure 1A). First, a so-called canonical binding site has been characterized that uses positively charged residues located between the β1-β2, β3-β4 and β6-β7 loops. Canonical binding sites have been reported for a variety of PH domains, such as those of PLCδ and b/akt [6], [8]. Second, a non-canonical binding site has been observed for the PH domain of β-spectrin, Tiam1 and ArhGAP9 that is formed between the β1-β2 and β5-β6 loops, adjacent to the canonical site but on the other side of the β1-β2 loop [17], [18], [19].

The PH domain-containing protein Slm1 from *S. cerevisiae* is a component of the target of rapamycin complex 2 (TORC2) signaling pathway. Slm1 plays a role in the regulation of actin organization [20] and is also a target of sphingolipid signaling during the heat shock response [10]. The PH domain of Slm1 is known to bind to PtdIns(4,5)P_2_ [20], and also PtdIns(4)P and a phosphorylated sphingolipid species, dihydrosphingosine 1-phosphate (DHS-1P) [12] (Figure S1). We have previously reported that *in vitro*, the Slm1-PH domain (Slm1-PH) interacts with liposomes containing PtdIns(4,5)P_2_ and DHS-1P cooperatively. Similarly, *in vivo* Slm1 association with specialized membrane microdomains, the eisosomes, requires the metabolism of both PtdIns(4,5)P_2_ and sphingolipids [12]. This led to the view that the recruitment of Slm1-PH to cellular membranes might involve a novel mechanism, in which not only PtdInsPs but also phosphorylated sphingolipids could act as anchoring ligands. We previously reported a structure for the apo Slm1-PH (at 2.0 Å) that depicted only part of the non-canonical binding site. This revealed that Slm1-PH might possess two binding sites for anionic lipids [12]. However, in the absence of a ligand-bound holo structure, the detailed modalities of ligand recognition remained largely elusive.

Here we report the crystal structure of the apo form of Slm1-PH domain at 1.76 Å resolution together with the holo Slm1-PH bound to different mimics of lipid head groups: i) a 1.68 Å resolution structure of Slm1-PH in complex with phosphoserine (surrogate of DHS-1P head group) and ii) 1.4 Å and 1.8 Å resolution structures of Slm1-PH in complex with inositol 4-phosphate (Ins(4)P; surrogate of the PtdIns(4)P head group). The results show that the non-canonical binding site of Slm1-PH is used for ligand binding, similar to the site described previously for β-spectrin, Tiam1 and ArhGAP9 [17], [18], [21] and which therefore suggests that this interaction mode has been conserved in yeast. Furthermore, our data also support the existence of an active canonical binding site in Slm1-PH, thus providing a structural model for the previously reported cooperativity between PtdInsPs and DHS-1P binding [12].

## Results and Discussion

### Ligand screening for the Slm1-PH domain

Previous experiments, based on isothermal titration calorimetry (ITC), have shown that Slm1-PH binds weakly to PtdIns(4,5)P_2_ or DHS-1P alone. Both lipids need to be present for the interaction to reach affinities of 1.8 μM [12], which is similar to those observed for other PH domain-ligand pairs such as PLCδ-PH and PtdIns(4,5)P_2_ (1.7 μM; [6]) or β-spectrin and PtdIns(4,5)P_2_ (40 μM; [18]). To understand the interactions and specificities of Slm1-PH in complex with surrogate lipid head groups, more than 50 crystallization experiments were screened that included both co-crystallization and soaking techniques. Seven different soluble compounds that mimic PtdInsPs and DHS-1P head groups (Figure S1) were used either alone or in combination. These included various inositol phosphates (InsPs), phosphoserine, the sphingosine analog FTY720 and its phosphorylated derivative, FTY720(P) [22], [23], [24] (Table 1). Only co-crystallization experiments with bound ligands produced acceptable diffraction data. The results presented here include: (i) the apo structure of Slm1-PH that we resolved at 1.76 Å resolution; (ii) one structure with phosphoserine at 1.68 Å resolution; and (iii) two structures of Slm1-PH in complex with Ins(4)P at 1.4 and 1.8 Å resolution (Tables 1 and 2).

**Table 1:** Crystallographic screening for Slm1-PH domain ligands.

Surrogate ligands	Result	Lipid
Apo Slm1-PH	1.76 Å resolution	Holo form
Ins(4)P	1.4 Å resolution; Ins(4)P bound	PtdIns(4)P
Ins(4)P	1.8 Å resolution; Ins(4)P bound	PtdIns(4)P
PS+Ins(1,4,5)P_3_	1.68 Å resolution; Phosphoserine bound	DHS-P + PtdIns(4,5)P_2_
PS+Ins(4)P	2.7 Å resolution; Phosphoserine bound	DHS-1P + PtdIns(4)P
FTY720(P)+Ins(4,5)P_2_	1.6 Å resolution; No bound ligand	DHS-1P + PtdIns(4,5)P_2_
Ins(3)P	1.7 Å resolution; No bound ligand	PtdIns(3)P
PS+Ins (3)P	1.6–1.8 Å resolution; No bound ligand	DHS-1P + PtdIns(3)P
PS+Ins(4,5)P_2_	2.0 Å resolution; No bound ligand	DHS-1P + PtdIns(4,5)P_2_
PS+Ins(1,4,5)P_3_	2.5 Å resolution; No bound ligand	DHS-1P + PtdIns(4,5)P_2_
Ins(1,4,5)P_3_	2.5 Å resolution; but only in one direction	PtdIns(4,5)P_2_
Ins(1,4,5)P_3_	2.6 Å resolution; No bound ligand	PtdIns(4,5)P_2_
PS+Ins(1,4,5)P_3_	3.1 Å resolution; No bound ligand	DHS-1P + PtdIns(4,5)P_2_
Ins(4,5)P_2_	3.4 Å resolution; No bound ligand	PtdIns(4,5)P_2_
PS+Ins(1,4,5)P_3_	4.5 Å resolution; No bound ligand	DHS-1P + PtdIns(4,5)P_2_
FTY720	Too mosaic diffraction to process so far	DHS
Ins(4)P+Ins(1,4,5)P_3_	No diffraction	PtdIns(4)P+ PtdIns(4,5)P_2_
FTY720(P)+Ins(4)P	No crystals	DHS-1P + PtdIns(4)P

* PS  =  phosphoserine.

* Crystals of the same protein-ligand complex were obtained in different crystallization conditions.

* FTY720  =  synthetic analog of sphingosine, currently studied as a potent immunosuppressive and immunomodulatory agent [22], [23], [24]; FTY720(P)  =  phophorylated FTY720.

**Table 2:** Data collection and refinement statistics.

	Apo	Phosphoserine and malonate	Ins(4)P		Ins(4)P (inverted phosphate group)
X-ray source	Synchrotron^a^	Synchrotron^b^	Synchrotron^a^		Synchrotron^a^
Detector	ADSC Q105 CCD @ ESRF	MAR225 @ SLS	ADSC Q105 CCD @ ESRF		ADSC Q105 CCD @ ESRF
Wavelength (Å)	0.97242	1.0000	1.06650		1.06650
Temperature (K)	100	100	100		100
Resolution (Å) at which data were collected	1.60	1.60	1.40		1.75
Data Statistics for refined atoms					
Space group	P21	P22121	P21		P21
No. of molecules in a.u.	4	2	4		4
Unit cell dimensions	37.3,82.3,76.4, β = 90.1	37.5,73.6.82.5, β = 90.01	37.0,71.6.82.7, β = 90.02		37.0,71.6.82.6, β = 90.1
R_meas_ c (%)	4.4 (30.0)	9.7 (53.5)	4.5 (53.2)		5.0 (35.0)
I/σ	26.02 (5.0)	10.6 (3.3)	16.6 (2.5)		18.7 (4.0)
Completeness (%)	95.6 (70.3)	98.6 (93.3)	95.6 (92.8)		99.2 (96.0)
Redundancy	6.6 (4.0)	5.3 (6.9)	4.6 (3.6)		3.7 (3.5)
Unique reflections	50454	26485	80522		39703
No. of reflections used	43737	26460	73073		37717
Refinement					
Resolution (Å)	1.76	1.68	1.45		1.80
R_work_, R_free_ d	18.8/22.7	21.4/24.1	19.2/22.6		18.6/24.0
No. of atoms					
Protein	3692	1739	3500	3543	
Water	357	180	380	273	
Ligand(s)	10	31	88	102	
B factors [average B value (Å^2^)for the refined atoms]					
Protein	27.5	31.3	23.0	21.7	
Water	35.9	37.9	36.5	33.2	
Ligand(s)	61.2	85.8	67.6	69.3	
R.m.s deviations					
Bond lengths (Å)	0.006	0.006	0.006	0.011	
Bond angles (°)	1.04	1.11	1.02	1.83	

* Highest resolution shell is shown in parenthesis.

a beam line ID29 at European Synchrotron Radiation Facility at Grenoble in France.

b PX beam line at Swiss Light Source, Paul Scherrer Institut 5232 Villigen PSI Switzerland.

c Rmeas is the redundancy-independent merging R-factor (intensities) *R*meas  = (Σ*h*(*n*/(*n*-1))^0.5^ Σ*j*|*Îh* -*Ihj*|)/(Σ*hjIhj*) with *Îh*  = (Σ*jIhj*)/*nj* Where *N* is the number of times a given observation has been observed (ie j = 1, n).

d 3% of the total reflections were excluded for cross-validation.

### Overall structure of the Slm1-PH domain

Although the sequence identity with other PH domains is rather low (∼18%), the overall fold of Slm1-PH is similar to previously determined structures of PH domains [6], [16], [17], [18], [25] (Figure 1A). The core of each monomer consists of seven antiparallel β-strands with a C-terminal amphipathic α-helix connected via a long and flexible loop. The loops connecting the β-sheets, especially β3-β4 and β5-β6 (residues 501-512 and residues 526-542, respectively), are long and constitute putative ligand-binding sites. The interior of the core consists of hydrophobic residues. Electron densities corresponding to the Ins(4)P molecules were visible between the β1-β2 and β5-β6 loop regions (difference density maps; Figure S2). In addition, all structures in complex with ligands show regions of additional density between the bottom of the β1-β2 loop and the N-terminus of Slm1-PH. Intriguingly, we could not assign any ligand to these extra densities. Interestingly for all four structures, much of the binding space in the canonical binding site is occupied by the β5-β6 loop of the neighboring molecule (Figure 1B and C). As the interactions between the two molecules involve the backbone of β1-β2 and no specific side-chain residue, this probably represents a crystallographic packing artifact. Compared with our previously published structure at 2 Å [12], our new apo structure clearly visualizes the full β5-β6 loop, which plays a key role in accommodating ligand molecules in the non-canonical binding site (see below).

### The Slm1-PH domain binds lipid via a non-canonical binding site

**Figure 2:**
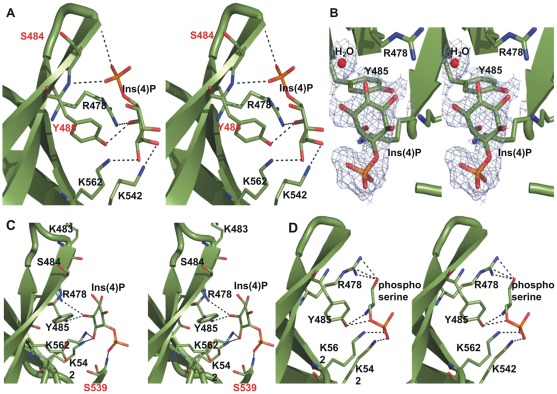
Detailed stereo diagrams of bound ligands. (**A**) Interactions made by Ins(4)P (green) and the residues at the non-canonical binding site of Slm1-PH (also in green). The phosphate group of Ins(4)P is making contacts with the β1-β2 region (residues involved are shown in red). Residues critically involved in the interactions are labeled by the single-letter code and shown in stick representation (see text for details). (**B**) The Ins(4)P is shown in the experimental 2Fo-Fc electron density map at 1.2 sigma level. The water molecule (red) corresponds to P1 position of Ins(1,4,5)P when docked into the non-canonical binding site. Position of the Ins(4)P is oriented to maximize the clarity of the figure. (**C**) Similar interactions are shown here with the Ins(4)P molecule for which the phosphate position is seen on the opposite side of the binding pocket, making contacts with residues of β5-β6 (again in red). (**D**) Stereo representation of phosphoserine bound in the non-canonical binding site. The dashed lines shown between atoms represent hydrogen bonds between ligand(s) and the residues at the non-canonical binding site of Slm1-PH.

In our complex crystal structures, all negatively charged ligands localize to the non-canonical binding site defined by the flexible loops β1-β2 and β5-β6, which form a wide and shallow pocket (Figure 1A). As described for other PH domains, the β1-β2 sheets and their connecting loop play a central role in ligand binding (Figure 2) [2], [5], [8], [11], [16], [17], [18], [26], [27]. For the two structures determined in complex with Ins(4)P, the positions of the inositol ring overlap. In both cases, the oxygen atoms of the inositol ring contact the β1-β2 region through the hydroxyl group of Tyr485 (hydrogen bond length  = 2.8 Å) and the NH_2_ group of Arg478 (hydrogen bond length  = 2.5 Å), and the β5-β6 loop through the ε-amino group of Lys542 (hydrogen bond length  = 2.7 Å). In addition, we observed a contact with the ε-amino group of Lys562 (hydrogen bond length  = 3.2 Å) from the β6-β7 strand. Interestingly, the two structures differ with respect to the orientation of the phosphate at position 4 (P4), which contacts either the β1-β2 loop or turns 180^°^ and contacts the β5-β6 loop (Figure 2A, C). In the first case, the main-chain nitrogen of Tyr485 and the carbonyl oxygen of Ser484 (hydrogen bond lengths  = 3.0 and 3.1 Å, respectively) of the β1-β2 loop region interact with P4 (Figure 2A), whereas in the other structure the main-chain nitrogen atom of Ser539 of the β5-β6 loop forms hydrogen bonds of 2.6 Å with the oxygen atom of P4 (Figure 2C).

The phosphoserine contacts the β1-β2 region and the β5-β6 loop through the same key residues as Ins(4)P (Figure 2D). Specifically, the hydroxyl group of Tyr485 hydrogen bonds with the nitrogen atom of phosphoserine (hydrogen bond length  = 2.9 Å) and the NH_2_ group of Arg478 interacts with the oxygen atom of phosphoserine (hydrogen bond length  = 2.8 Å). In addition, the ε-amino group of Lys542 from the β5-β6 loop and the ε-amino group of Lys562 from the β7-strand also interact with the phosphate group of the phosphoserine molecule (hydrogen bond lengths  = 2.4 Å and 2.7 Å, respectively).

**Figure 3:**
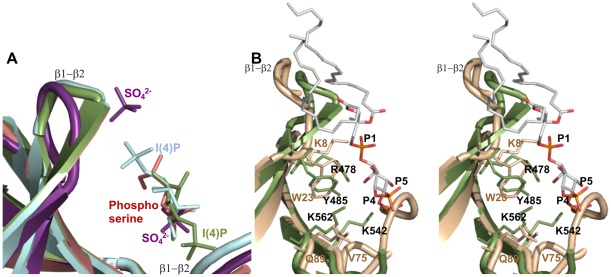
Superposition of Slm1-PH domain structures. (**A**) The different ligands (sulfate (violet), phosphoserine (salmon), Ins(4)P (green) and also the turned-over Ins(4)P (light blue)) are occupying the same non-canonical binding pocket, confirming the readily available large binding site of the Slm1-PH. (**B**) Stereo diagram of PtdIns(4,5)P_2_ (atom colors) modeled in the non-canonical binding site of Slm1-PH (green) and overlaid onto the β-spectrin PH domain (wheat color). This shows the conservation of side chains that contact the ligands.

Overall, the ligands show very similar binding modalities and when superposed on each other, they all share common positions in the non-canonical binding site that overlap with previously observed positions of sulfate ions in the holo form of Slm1-PH (Figure 3A). In the non-canonical binding site there are four residues that are consistently involved in ligand binding: Arg478, Tyr485, Lys542 and Lys562. Of these, Arg478 is conserved among PH domains that contain non-canonical binding sites. Interestingly, Arg478 has been shown to be important for ligand binding *in vivo* by mutation analysis [12]. We subsequently docked PtdIns(4,5)P_2_ into the non-canonical binding site of Slm1-PH using the structure of β-spectrin in complex with Ins(1,4,5)P_3_ [18]. The Ins(4)P and phosphoserine molecules superposed well on to the Ins(1,4,5)P_3_ of the β-spectrin PH complex positioned in the non-canonical binding site. The resulting model shows that the PtdIns(4,5)P_2_ fits well within the binding site, making contact with all conserved residues (Figure 3B). This again suggests a key role for residues Arg478, Tyr485, Lys542 and Lys562 in building a positively charged array that is essential for the correct orientation and anchoring of the PH domain to PtdIns(4,5)P_2-_containing membranes. Overall, our results are consistent with the view that the yeast Slm1-PH belongs to the class of PH domains that bind ligands through a non-canonical binding site. Therefore, this type of PH domain is not restricted to higher eukaryotes, and has been conserved from yeast to mammals.

**Figure 4:**
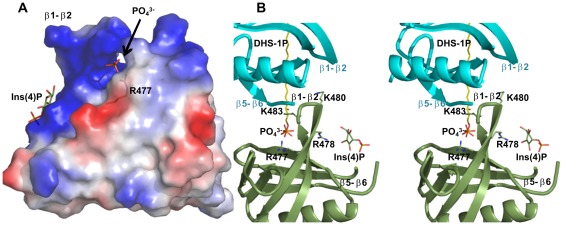
Surface charge distribution. (**A**) The Slm1-PH provides a positively-charged cavity to interact with a negatively charged Ins(4)P molecule (in green). The back of the β1-β2 region (by rotating Slm1-PH by 180 degrees) shows a more positively charged region. In several structures of Slm1-PH determined in complex with ligand analogs, we built a phosphate group or full Ins(4)P at this additional positively charged site (see text for details). The Arg477 side chain that turns towards the canonical binding site is also shown in transparent stick format. (**B**) Stereo diagram of Slm1-PH (green) showing Arg477, Arg478, Lys480 and Lys483 on either side of the β1-β2 strands in the vicinity of the negatively charged residues of the β5-β6 loop of the neighboring molecule (cyan). Part of the β5-β6 loop is truncated for the clarity of the figure. The bound phosphate group is shown in stick representation at the back of the β1-β2 region towards the canonical binding site. We have modeled the natural ligand (DHS-1P in yellow) aligning with the phosphate position in this region. Ins(4)P is also shown in the non-canonical binding site.

**Figure 5:**
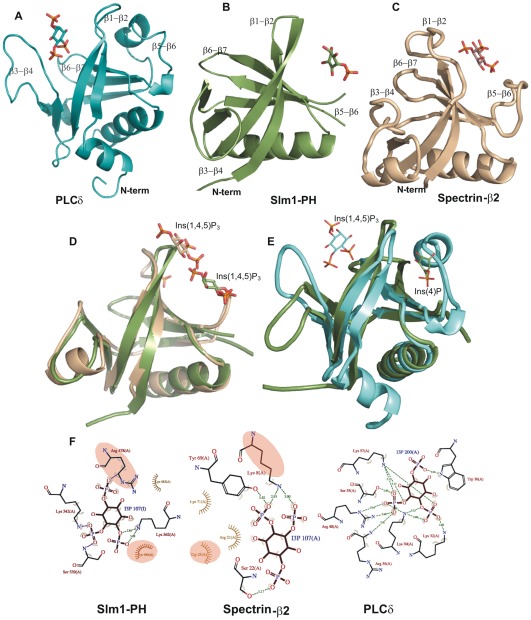
Comparison of canonical and non-canonical binding sites. (**A**) Structure of the PLCδ (1MAI.PDB) in steel blue with bound Ins(1,4,5)P_3_ in the canonical binding site, which is formed by β1-β2, β3-β4 and β6-β7 loop regions; (**B**) Cartoon representation of Slm1-PH in green, Ins(4)P is bound in the non-canonical binding site; (**C**) Structure of the β-spectrin PH domain (1BTN.PDB) in wheat color with bound Ins(1,4,5)P_3_ again in the non-canonical binding site. (**D**) Superposition of the Slm1-PH (green) modeled with Ins(1,4,5)P_3_ onto the β-spectrin PH domain (wheat) and (**E**) onto PLCδ (cyan) (**F**) LIGPLOT representation [38] showing residues involved in ligand binding in the non-canonical binding site (Slm1-PH and β-spectrin) and the canonical binding site (PLCδ). The conserved residues between PH domains of Slm1 and β-spectrin involved in ligand binding are highlighted in pale yellow.

**Figure 6:**
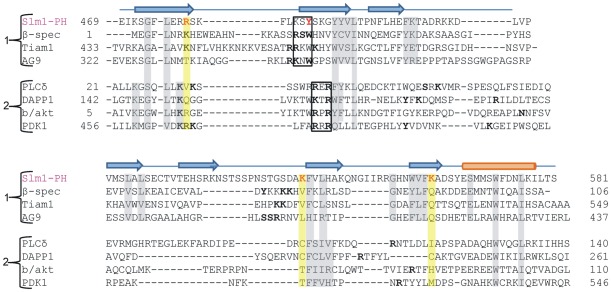
Structure-based sequence alignment of PH domains of Slm1, β-spectrin and PLCδ. The alignment was produced using the PROMALS3D program [39] and corrected manually on the basis of the three-dimensional structure of the Slm1-PH. The matched-up sequences were from RCSB/PDB. The secondary structure elements revealed in the Slm1-PH crystal structure are shown above the sequence: β-sheets are shown by blue arrows and the C-terminal helix by an orange cylinder. The residues of Slm1-PH involved in Ins(4)P interaction are shown in red. The residues highlighted in yellow are aligned with the ligand-interacting residues of the Slm1-PH. Residues of different classes of PH domains that are involved in ligand binding are shown in ‘bold’. The R/KXR and R/KXW motifs for ligand binding in the canonical and non-canonical binding sites respectively, are shown in the black box. The structure-based sequence alignment showing overall conserved or semi-conserved residues are represented in grey. Sequences are grouped according to ligand binding in the non-canonical and canonical binding pockets.

### Potential binding sites for a second anionic lipid head group

It has been shown that *in vitro*, Slm1-PH binds to both PtdInsPs (PtdIns(4)P, PtdIns(4,5)P_2_) [9] and DHS-1P in a cooperative manner [12]. We therefore proposed that the Slm1-PH contains both non-canonical and canonical binding sites. Consistent with this hypothesis, our structure here reveals ligand bound in the non-canonical binding site and an additional positively charged region in the canonical binding site that remains available for binding to additional negatively charged head groups (Figure 4A). This encompasses the Arg477, Lys480 and Lys483 residues that point towards the canonical binding site and are important for the recruitment of Slm1 to biological membranes *in vivo* [12]. Interestingly, we also observed extra electron densities next to the canonical binding site that could be filled with a phosphate group (Figure 4 and Figure S3A). However, the identity of the ligand could not be unambiguously determined. Crystallization conditions did not contain any phosphate ion but the buffer was mildly acidic (pH 6.5), which could lead to partial hydrolysis. This could become significant given the high concentration of ligand used (10-fold excess over Slm1-PH) and the long incubation time (one week) required to obtain optimal crystals. Another possibility is that the full Ins(4)P bound, but the electron density for the inositol ring was too poor to be built. Consistent with the latter, we observed that by slightly loosening the restraints, a region of density in continuation with the phosphate group emerged in one out of four molecules in the asymmetric unit. We could fit an inositol ring in this region, even though the densities for some of the hydroxyl groups were missing at 1 sigma level (Figure S3B). This phosphate group lies in the cavity formed by His557, His546, Trp559, Val544 and Phe481 of the neighboring molecule. The oxygen atoms of the phosphate group interact with the ε-amino group of positively charged Lys480 (bond length  = 3.2 Å), ND_1_ of His557, the ND_2_ atom of Asn558, and the main-chain nitrogen atom of Asn558 (bond lengths  = 2.6 Å, 2.9 Å and 2.4 Å respectively) through hydrogen bonds (Figure S3A). We observed that the long and flexible side-chain of Arg477 points towards the phosphate group (bond length  = 8.5 Å) (Figure 4B) and the two could be modeled as close as 3 Å (Figure S3C). Our structures show that, along with other residues, Arg477 and Arg478 contribute to ligand binding in both of the two putative binding sites (Figure 4B and Figure S4). This is consistent with previous analyses, which showed that mutation of these residues to alanine either alone or in combination caused defects in Slm1 function and its ability to associate with biological membranes ([12] and Figure S4). The stronger and apparently cumulative effect of the double mutant supports the view that binding to both sites is required for Slm1 function. Taken together, our results clearly suggest the existence of an additional positively charged binding region adjacent to the non-canonical binding site.

### Comparison of Slm1-PH domain with other PH domains

Several structures of different PH domains have been determined using X-ray crystallography or nuclear magnetic resonance (NMR) spectroscopy [18], [27], [28], [29], [30]. Many of these are in complex with inositol phosphate ligand bound in either the canonical or the non-canonical binding site (Figure 5). Irrespective of the binding scenario (canonical or non-canonical), a series of positively charged residues within the β1-β2 loop region are relatively conserved (Figure 6; black box). A structure-based sequence alignment with other PH domains reveals that Slm1-PH is most similar to the PH domains of β-spectrin, Tiam1 and ArhGAP9, which bind to ligands through their non-canonical sites. The superposition of Slm1-PH onto the PH domain of β-spectrin shows the conserved β1-β2 loop region accommodating the ligand in the non-canonical binding site (Figure 5D and F). Indeed, Slm1-PH lacks the characteristic KXK/RXR motif in the β1-β2 loop that is present in PH domains with a canonical binding site (b/akt, Btk, Grp1 and PLCδ [5], [6], [8], [18], [19] (Figure 5E and F). Also in Slm1-PH, the most conserved positively charged residue within the β1-β2 loop (Arg478) is flipped towards the non-canonical binding site to interact with the Ins(4)P molecule. In PLCδ, a small valine residue occupies a similar position and also points towards the non-canonical binding site. This is consistent with the fact that the space between the β1-β2 and the β5-β6 loops is very narrow, which excludes the non-canonical binding mode in PLCδ (Figure S4).

Similar to β-spectrin, Tiam1 and ArhGAP9, the β1-β2 loop of Slm1-PH contains a conserved R/KXW motif, which is involved in ligand binding at the non-canonical binding site. The only minor difference is that the tryptophan is replaced by another aromatic residue, Tyr485. We also observe interesting differences between Slm1-PH and β-spectrin, Tiam1 and ArhGAP9 that may account for the dual binding specificity of Slm1-PH. For example, a non-conserved positively charged residue (Lys542) participates in ligand binding in the Slm1 PH, whereas a series of positively charged residues are absent within the β5-β6 loop region. Finally, in the case of Slm1-PH, ligand binding is also supported by Lys562 of the β6-β7 region (Figure 6), which is not conserved in other PH domains. These differences are consistent with the hypothesis that Slm1-PH may have altered binding properties compared with previously characterized PH domains.

In summary, here we present the first three-dimensional structures of the Slm1-PH bound to Ins(4)P and phosphoserine and show that the Slm1-PH exhibits a non-canonical PtdInsPs-binding mechanism. The non-canonical binding site is shallow, large and solvent-exposed, and so the phosphate position of Ins(4)P in one of the structures was seen on the opposite side of the molecule. The non-canonical binding site accommodated anions from the crystallization conditions, and also the phosphoserine molecule. The long β5-β6 loop occupied part of the space at the canonical binding cavity, making it less available for ligand binding. We nevertheless observed a large positively charged region behind the β1-β2 loop towards the canonical binding site that associated with a phosphate group. This putative binding surface could accommodate a second ligand and account for the cooperative binding described for the Slm1-PH.

## Materials and Methods

### Expression, purification and crystallization

The Slm1-PH was expressed from *Escherichia coli* and purified using affinity chromatography. Cloning, expression and purification of the recombinant protein used in this study were performed using the methods described previously [12].

### Crystallization of the Slm1-PH domain

Initial apo crystals of the Slm1-PH were obtained through a screen with the mosquito crystallization robot (TTP Labtech) using the sitting drop vapor diffusion technique in 96-well plates with drop and reservoir volumes of 0.2 μl and 100 μl, respectively. Crystals were routinely grown in 24-well plates (Hampton Research) at 15°C by the hanging drop vapor diffusion technique. Equal volumes (0.75 μl/0.75 μl) of protein (15 mg/ml) and crystallization buffer (0.1 M MES + 30% PEG5000 + 0.2 M ammonium sulfates, pH 6.5) were mixed on a cover slip, which was subsequently equilibrated against a reservoir containing 500 μl of crystallization buffer. Micro-seeding was used to obtain larger single crystals. The optimal hexagonal crystals grew in about eight days to a size of approximately 100 μm × 100 μm × 50 μm. Subsequently, sulfate ions were replaced with malonate in the crystallization conditions (0.1 M MES + 28% PEG5000 + 0.2 M sodium malonate, pH 6.5) to avoid interference with ligand binding.

Co-crystallization and soaking techniques were used to obtain complex crystals. Several crystals of Slm1-PH were obtained with and without head group analogues of PtdInsPs and lipids (Table 1). Crystals of Slm1-PH in complex with Ins(4)P were obtained by using 10-fold molar excess of Ins(4)P (Sigma Aldrich). Monoclinic rod-like crystals formed in about a week and grew to approximately 150 μm x 90 μm x 300 μm from the crystallization conditions containing 0.1 M Bis-Tris + 25% PEG3350 + 0.2 M sodium malonate, pH 5.5. All crystals were harvested by transfer to a cryo-protectant solution of 15% ethylene glycol in the mother liquor, rapidly collected in a nylon loop (Hampton Research, USA) and flash-cooled in liquid nitrogen.

### Data collection, structure determination and refinement

Data were collected at various synchrotron beamlines at the European Synchrotron Radiation Facility (ESRF) in Grenoble, France and the Swiss Light Source (SLS) in Villigen, Switzerland. All the data sets were processed and scaled using the XDS program package [31]. For all the structures presented here, the phase problem was solved by molecular replacement [32] using the program PHASER [33] and the coordinates of apo Slm1-PH (3NSU; [12]) as a search model. Two flexible loops (residues: 501–512 and 526–542) of the search model were truncated for the phase solution. The model was refined in alternating cycles of refinement with PHENIX [34]. Manual rebuilding and visual comparisons were made using the graphical program COOT [35].

The stereo chemical quality of the final model was assessed using PROCHECK [36]. Molecular diagrams were drawn using the *PyMOL* molecular graphics suite [37]. Details of the diffraction data collection, quality and refinement statistics are given in Table 2.

### Protein databank accession number

The atomic coordinates and structure factor amplitudes have been deposited in the Protein Data Bank Europe, http://www.ebi.ac.uk/pdbe/ [PDB ID codes: 4A5K, 4A6F, 4A6H and 4A6K].

## Supporting Information

Figure S1 **Chemical drawings of the physiological ligands of the Slm1-PH.** The head-group mimics, Ins(4)P and phosphoserine, are indicated by red circles.
(TIF)

Figure S2 **Experimental Fo-Fc electron density map prior to modeling of a bound Ins**(**4**)**P molecule, contoured at 2.5** **sigma level.** Ins(4)P molecule and the residues involved in the interaction are shown in the stick format and labeled accordingly.
(TIF)

Figure S3 **Phosphate positioned in the cavity at the back of the β1-β2 region.** (**A**) Interaction with the residues of the β1-β2 and β6-β7 loops. The phosphate group and the residues involved in the interaction are shown in ball-and-stick format in the ribbon diagram of Slm1-PH in green. All the hydrogen bonds are shown by dashed lines. (**B**) Cartoon representation of Slm1-PH with Ins(4)P bound at the back of the β1-β2 loop region in one out of four molecules in the asymmetric unit. (**C**) Here we modeled/rotated the side chain of Arg477 (already facing towards the phosphate of Ins(4)P) that can come to within 3 Å of the phosphate group.
(TIF)

Figure S4 **Superposition of the conserved Arg477 and Arg478 of Slm1-PH** (**green**) **onto the residues Lys and Val of PLCδ** (**teal color**) **and Arg and Lys of β-spectrin** (**wheat color**) **PH domains.** (B) Impact of Arg477 and Arg478 mutation to alanine on Slm1 recruitment to biological membranes and Slm1 function *in vivo*, i.e. yeast growth and actin polarization. Adapted from [12].
(TIF)
