# Supplementary figures and images for: Structural Analyses of the Slm1-PH Domain Demonstrate Ligand Binding in the Non-Canonical Site
Source: PLoS One. 2012 May 4;7(5):e36526. doi: 10.1371/journal.pone.0036526 (PMC3344901; doi:10.1371/journal.pone.0036526)

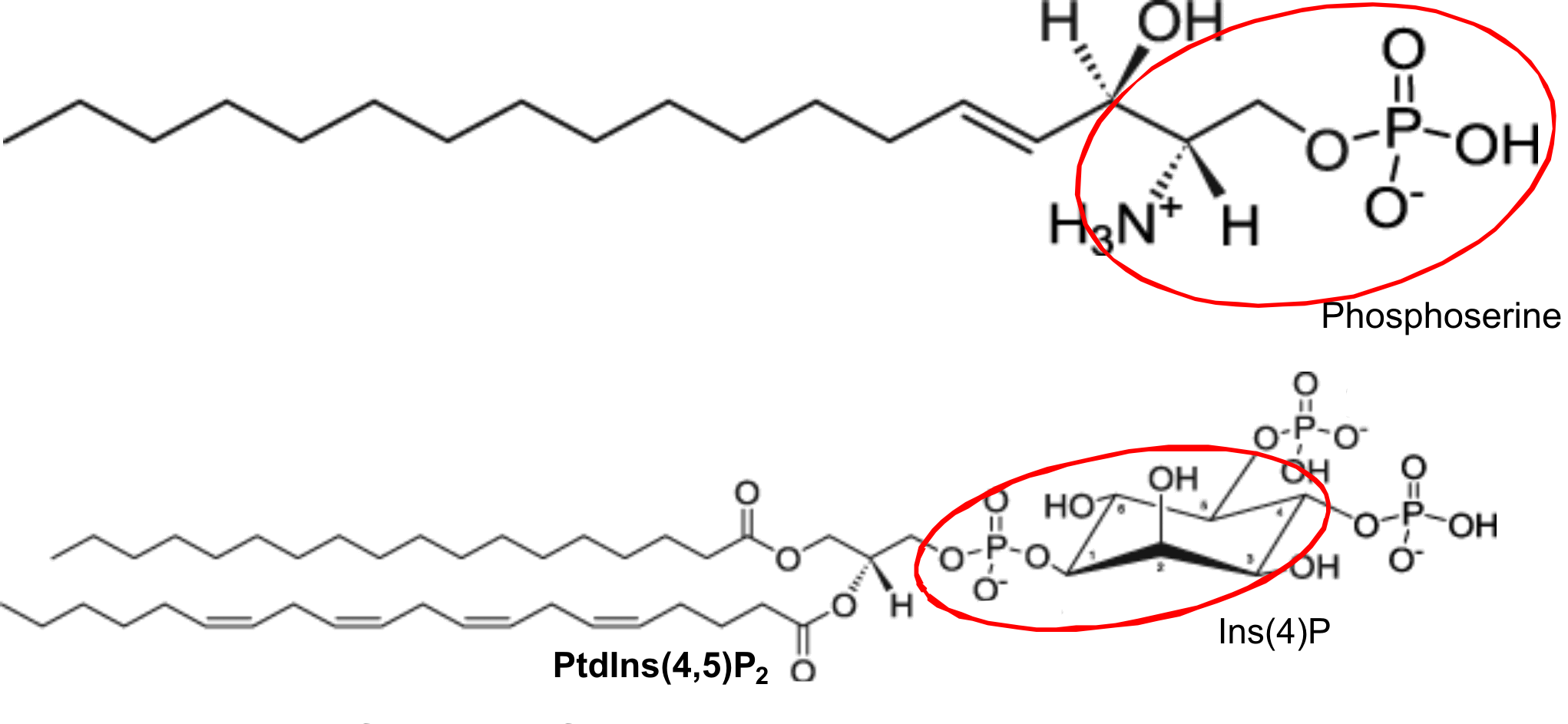

Supplement: Figure S1 — Chemical drawings of the physiological ligands of the Slm1-PH. The head-group mimics, Ins(4)P and phosphoserine, are indicated by red circles. (TIF) [file pone.0036526.s001.tif]

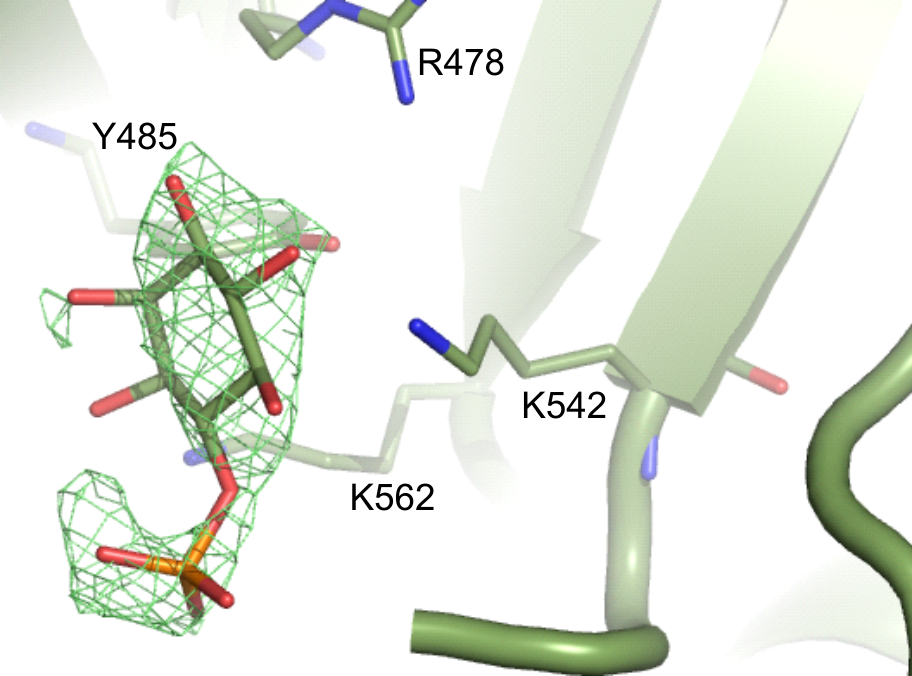

Supplement: Figure S2 — Experimental Fo-Fc electron density map prior to modeling of a bound Ins(4)P molecule, contoured at 2.5 sigma level. Ins(4)P molecule and the residues involved in the interaction are shown in the stick format and labeled accordingly. (TIF) [file pone.0036526.s002.tif]

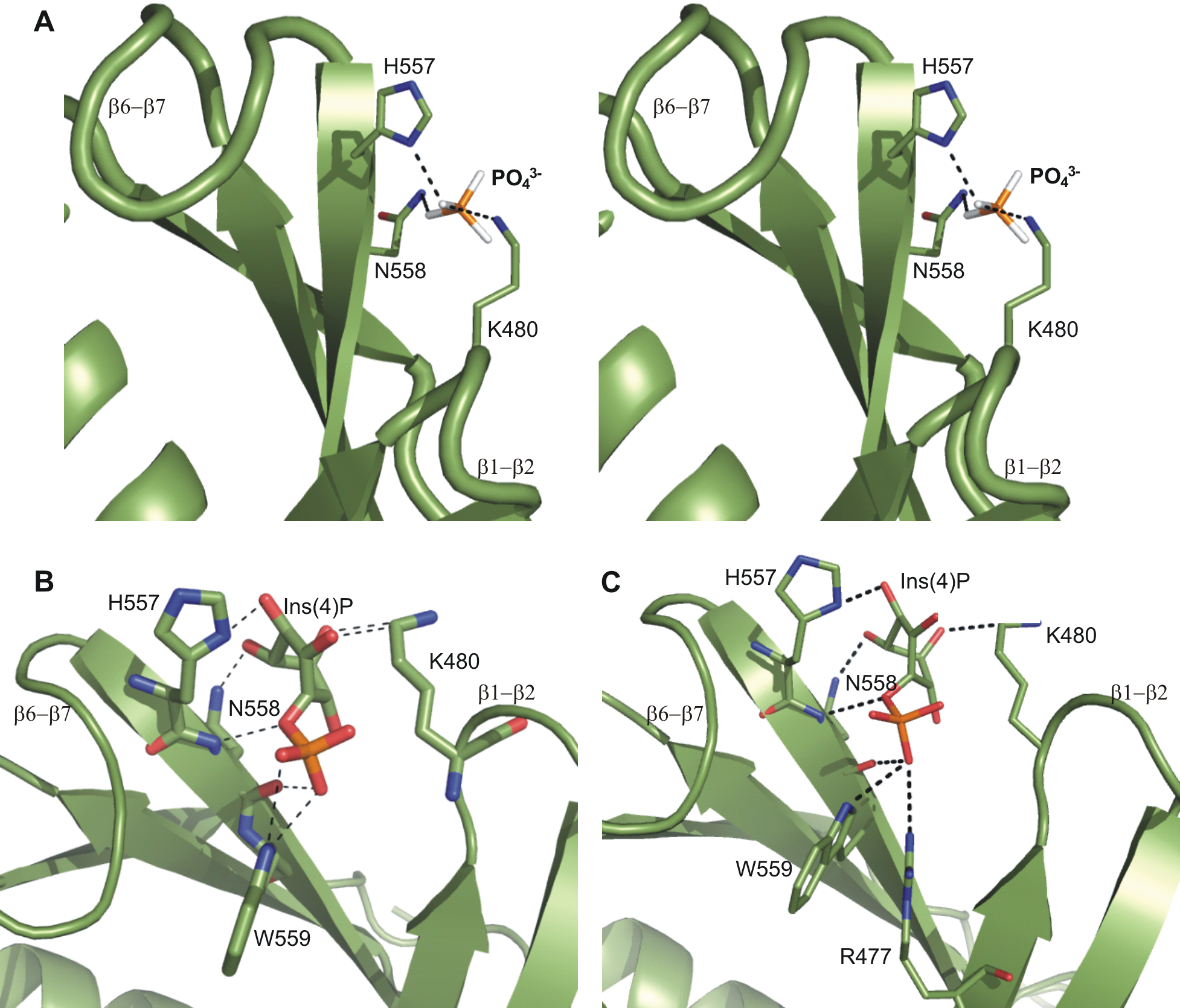

Supplement: Figure S3 — Phosphate positioned in the cavity at the back of the β1-β2 region. (A) Interaction with the residues of the β1-β2 and β6-β7 loops. The phosphate group and the residues involved in the interaction are shown in ball-and-stick format in the ribbon diagram of Slm1-PH in green. All the hydrogen bonds are shown by dashed lines. (B) Cartoon representation of Slm1-PH with Ins(4)P bound at the back of the β1-β2 loop region in one out of four molecules in the asymmetric unit. (C) Here we modeled/rotated the side chain of Arg477 (already facing towards the phosphate of Ins(4)P) that can come to within 3 Å of the phosphate group. (TIF) [file pone.0036526.s003.tif]

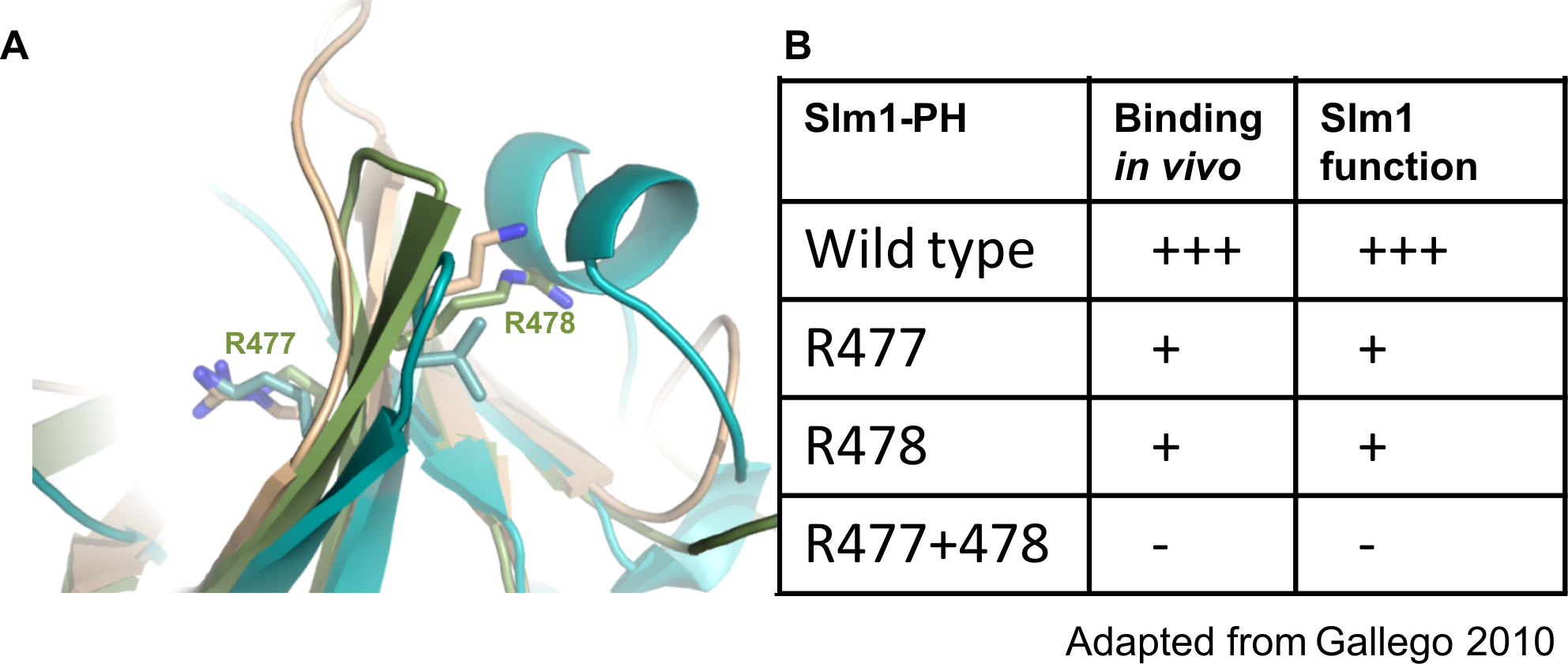

Supplement: Figure S4 — Superposition of the conserved Arg477 and Arg478 of Slm1-PH (green) onto the residues Lys and Val of PLCδ (teal color) and Arg and Lys of β-spectrin (wheat color) PH domains. (B) Impact of Arg477 and Arg478 mutation to alanine on Slm1 recruitment to biological membranes and Slm1 function in vivo, i.e. yeast growth and actin polarization. Adapted from [12]. (TIF) [file pone.0036526.s004.tif]
